# The prognostic significance of nutritional status using malnutrition universal screening tool in patients with pulmonary tuberculosis

**DOI:** 10.1186/1475-2891-12-42

**Published:** 2013-04-08

**Authors:** Shigeru Miyata, Mikio Tanaka, Daizo Ihaku

**Affiliations:** 1 Department of Internal Medicine, Hanna Hospital, 1-1-31 Terakawa, Daito, Osaka 574-0014, Japan; 2 Department of Pharmacy, Hanna Hospital, 1-1-31 Terakawa, Daito, Osaka 574-0014, Japan; 3 Department of Health Care Medicine, Kawasaki Medical School, 577 Matsushima, Kurashiki, Okayama 701-0192, Japan

## Abstract

**Background:**

Malnutrition is frequently observed in patients with pulmonary tuberculosis (TB). The present study aimed to examine the relationship between nutritional status using Malnutrition Universal Screening Tool (MUST) and the mortality of patients with pulmonary TB.

**Methods:**

Fifty-seven patients with pulmonary TB were analyzed. Nutrition assessment was carried out using MUST. The Cox proportional hazard model was applied to assess the ability of MUST to predict prognosis. Receiver operating characteristic curve analysis was used to assess MUST score as a prognostic indicator in pulmonary TB patients. To obtain optimal cut-off values for MUST score for prognostic assessment in TB patients, we used the maximum Youden Index.

**Results:**

For predicting the risk of mortality, the optimal cut-off value for MUST score was 3.5. Univariate and multivariate analyses identified age and MUST score ≥ 4 as significant independent prognostic factors for survival. The patients with MUST score ≤ 3 had a median survival of 481 days (95% CI: 453 to 510) and that for the patients with MUST score ≥ 4 was 304 days (95% CI: 214 to 394); the difference was statistically significant (P = 0.001).

**Conclusion:**

MUST appears to be a reliable tool for nutritional risk assessment of patients with pulmonary TB. In addition, MUST may be a useful prognostic indicator of survival in patients with pulmonary TB.

## Background

Tuberculosis (TB) has afflicted mankind from time immemorial. TB is also a reemerging infectious disease that was declared a global public health problem by the World Health Organization in 1993. In 2011, there were an estimated 8.7 million new cases of TB (13% co-infected with HIV) and 1.4 million people died from TB, including almost one million deaths among HIV-negative individuals and 430 000 among people who were HIV-positive. TB is one of the top killers of women, with 300 000 deaths among HIV-negative women and 200000 deaths among HIV-positive women in 2011 [1].

In Japan, the TB incidence rate fell below 20 per 100,000 in 2007 and continued to decline, reaching 19.0 in 2009. However, 24,170 TB patients were newly identified in 2009. In 2009, TB incidence rates of elderly populations aged 65–74, 75–84 and 85 or older were 26.5, 63.4 and 98.1 per 100,000 in Japan, respectively [2].

Aging of the population and the increased use of immunosuppressive treatments (e.g. cancer chemotherapy and immunomodulatory agents) highlight the need for additional strategies to maintain and improve TB control [3].

Malnutrition is frequently observed in patients with pulmonary TB. Several studies reported that patients with active pulmonary TB are malnourished, as indicated by reductions in the level of visceral proteins, anthropometric indexes and micronutrient status [4,5]. Nutrition support is necessary for malnourished TB patients. Nutrition risk assessment is important in providing nutrition support. The Malnutrition Universal Screening Tool (MUST) is a simple, quick, valid and reliable tool to identify patients who are malnourished or at risk of malnutrition.

To the best of our knowledge, no studies have examined the relationship between MUST and the mortality of patients with pulmonary TB. The objective of this study is to examine the relationship between MUST and the prognosis of patients with pulmonary TB.

## Methods

### Patients

Fifty-seven patients with pulmonary tuberculosis were analyzed. This is a retrospective study. This study was carried out in only one hospital (Hanna Hospital). The diagnosis of pulmonary TB was made on the basis of symptoms, chest radiographic infiltrates and the presence of *M. tuberculosis* on sputum culture. Patient age ranged from 17 to 91 years (mean, 64.9 years). Patient characteristics are summarized in Table 1. This study was approved by the institutional review board. This study was carried out in compliance with the Helsinki Declaration.

**Table 1:** Characteristics of 57 patients with pulmonary tuberculosis

**Characteristics**		**Values**
Gender (male/female)		42 (73.7)/15 (26.3)
Age (years)		64.9 ± 20.1
Body mass index (kg/m^2^)		19.3 ± 2.8
Underlying disease		
	Malignant disease	3 (5.3)
	Diabetes mellitus	8 (14.0)
Presence of hypoxemia on admission		4 (7.0)
Smear positive		50 (87.7)
Radiological findings		
	bilateral involvement	40 (70.2)
	cavitation	21 (36.8)
	effusion	10 (17.5)
Laboratory findings		
	Total Protein (g/dL)	6.6 ± 1.0
	Albumin (g/dL)	3 ± 0.8
	Cholinesterase (U/L)	136.1 ± 77.2
	Hemoglobin (g/dL)	12.0 ± 2.2
	CRP (mg/dL)	6.6 ± 6.0

Data are presented as n (%) or mean ± SD.

*TB*: tuberculosis.

### Anti-tuberculosis therapy

All patients received anti-tuberculosis therapy. All patients were in-patients at the start of anti-tuberculosis therapy. The regimen comprised isoniazid, rifampicin, ethambutol and pyrazinamide daily for two months, followed by isoniazid and rifampicin daily for the next four months. Dosage of anti-tuberculosis drugs by body weight is described in Table 2. Pyrazinamide was not administered to patients aged 80 years or older. The anti-tuberculosis therapy to patients aged 80 years or older was carried out for nine months.

**Table 2:** Dosage of anti-tuberculosis drugs

**Body weight (kg)**	**Isoniazid (mg)**	**Rifampicin (mg)**	**Ethambutol (mg)**	**Pyrazinamide (mg)**
30 < BW ≤ 40	200	300	500	1200
40 < BW ≤ 55	300	450	750	1200
55 < BW	300	600	750	1500

*BW*, Body weight.

*MUST*: Malnutrition Universal Screening Tool.

### Nutrition assessment

The nutrition assessment was carried out using MUST. MUST includes three variables: unintentional weight loss score in the preceding 3 to 6 months (weight loss < 5% = 0, weight loss 5–10% = 1, weight loss > 10% = 2), body mass index (BMI) score (BMI >20.0 = 0, BMI 18.5–20.0 = 1, BMI < 18.5 = 2) and acute disease effect score (Add a score of 2 if there has been or is likely to be no nutritional intake for >5 days). Each is scored on a scale of 0, 1 or 2 and their sum categorizes the malnutrition risk as low (0), medium (1) or high (≥ 2).

### Statistical analysis

Results are expressed as mean ± standard deviation. The Cox proportional hazard model was applied to assess the ability of MUST to predict prognosis. Receiver operating characteristic (ROC) curve analysis was used to assess MUST score as a prognostic indicator in pulmonary TB patients. Follow up was measured from the date of the first patient visit to the hospital until death or the last date of observation. Minimum follow-up duration was 90 days. The Kaplan-Meier method with the log-rank test was used to calculate survival. *P* values <0.05 were considered to be statistically significant. Statistical analysis was performed using SPSS 18.0 (SPSS, Inc., an IBM Company, Chicago, IL, USA).

## Results

**Figure 1:**
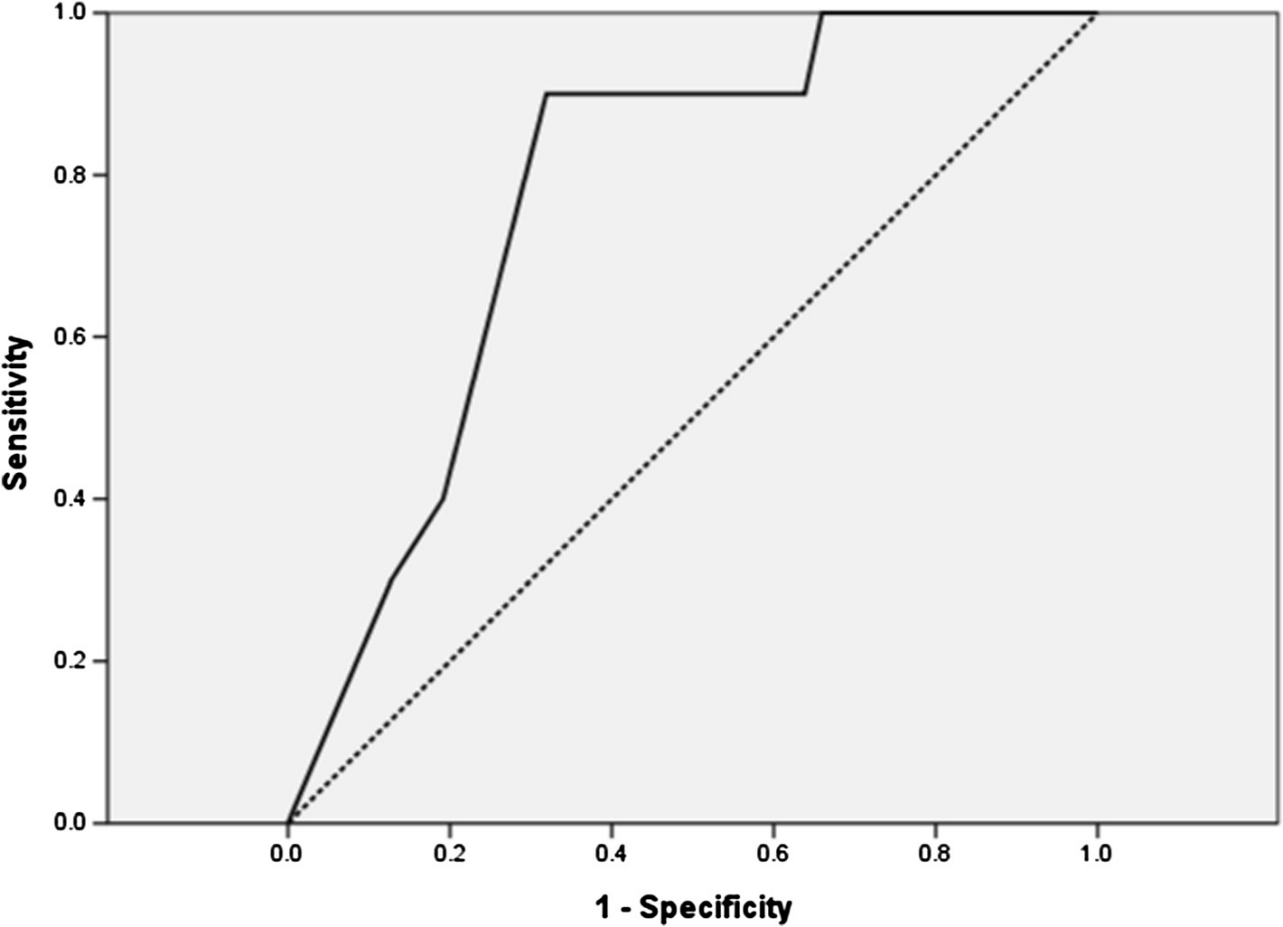
**Receiver operating characteristic (ROC) curve for MUST score.** Detailed legend: n = 57, AUC = 0.772 (95% confidence interval 0.636–0.908).

**Figure 2:**
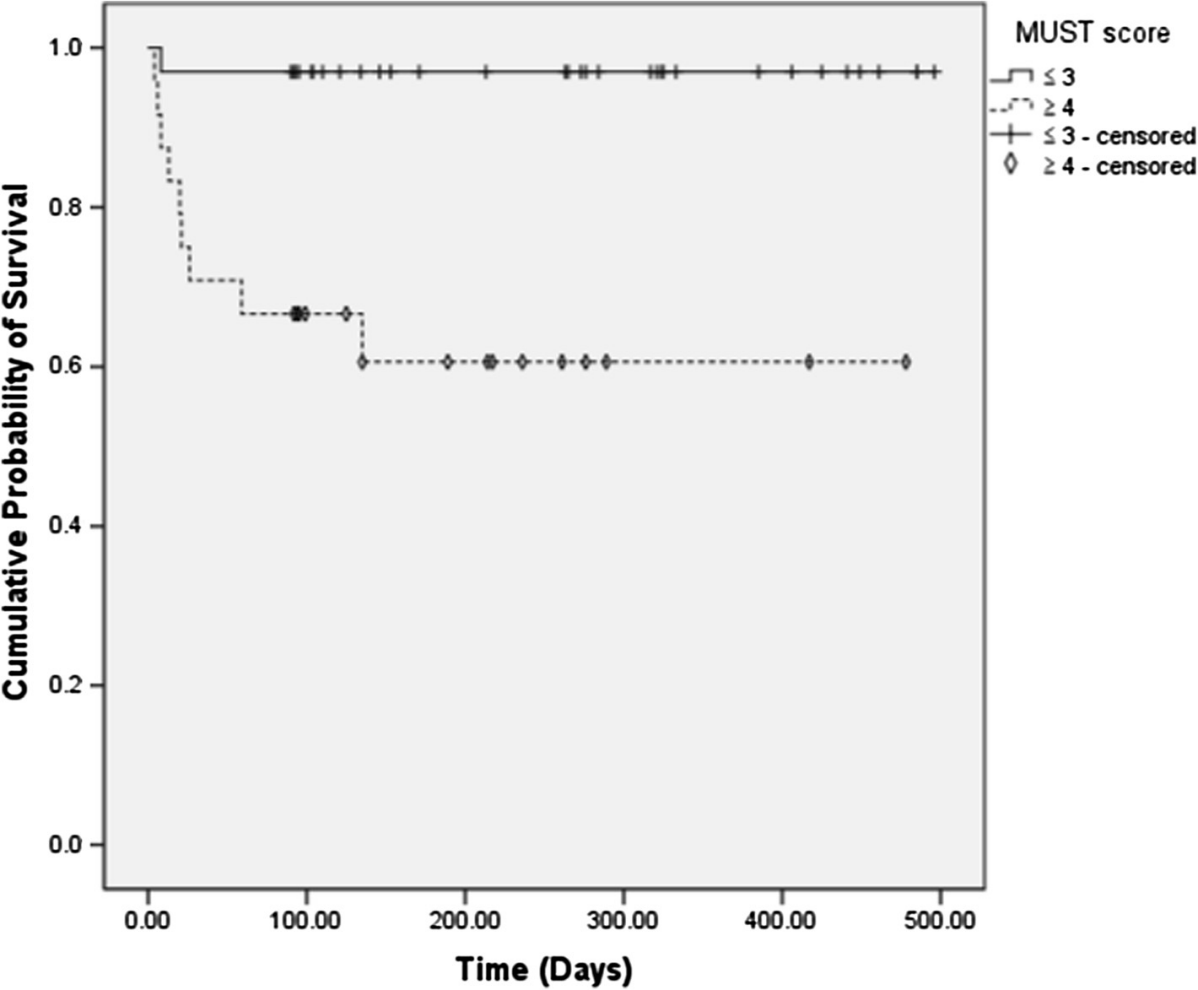
**Survival curves for the patients with MUST score ≤ 3 and the patients with MUST score ≥ 4.** Detailed legend: Censored patients were those who reached the end of their follow up without dying. Log-rank test, P = 0.001.

Table 1 summarizes the demographic and baseline clinical characteristics of the patients. There were 42 men and 15 women. The age ranged from 17 to 91 years (mean ± SD, 64.9 ± 20.1 years). Univariate and multivariate analyses showed that age was a significant prognostic factor. In contrast, high risk (MUST score ≥ 2) was not a significant prognostic factor (Table 3). An ROC curve was generated for further analysis of the prognostic value of MUST score, as presented in Figure 1. The area under the curve (AUC) was 0.772 (95% confidence interval 0.636–0.908). MUST score was considered to have moderate prognostic accuracy. To obtain optimal cut-off values for MUST score for prognostic assessment in TB patients, we used the maximum Youden Index. For predicting the risk of mortality, the optimal cut-off value for MUST score was 3.5. Using a cut-off value for MUST score of 3.5, the sensitivity was 90.0% and the specificity was 68.1% for survival. Patients were divided into two groups based on MUST score ≤ 3 or ≥ 4. On the basis of this cut-off value, the Cox proportional hazard model was applied to assess the ability of MUST score ≥ 4 to predict the prognosis of TB patients. Univariate analysis showed that age, albumin and MUST score ≥ 4 were significant prognostic factors (Table 3). Multivariate analysis was performed with a forward stepwise approach in age, albumin and MUST score ≥ 4. Multivariate analysis identified age (HR = 1.088, 95% CI = 1.017–1.165, P = 0.015) and MUST score ≥ 4 (HR = 0.097, 95% CI =0.012–0.777, P = 0.028) as significant independent prognostic factors for survival (Table 4). Figure 2 shows the survival curves for the patients with MUST score ≤ 3 and those with MUST score ≥ 4. The patients with MUST score ≤ 3 had a median survival of 481 days (95% CI: 453 to 510) and that for the patients with MUST score ≥ 4 was 304 days (95% CI: 214 to 394); the difference was statistically significant (P = 0.001). An HIV test was performed in no patient. Four patients underwent intravenous hyper-alimentation and three patients underwent respiratory management during the treatment.

**Table 3:** Univariate Cox proportional hazard regression analyses associated with survival

**Variables**		**HR**	**95% CI**	**P value**
Gender (female)		3.119	0.900–10.804	0.073
Age (years)		1.105	1.023–1.192	0.011
Body mass index (kg/m^2^)		0.862	0.680–1.093	0.22
Underlying disease				
	Malignant disease	1.953	0.247–15.455	0.526
	Diabetes mellitus	0.678	0.086–5.352	0.712
Presence of hypoxemia on admission		1.549	0.196–12.255	0.678
Smear positive		1.307	0.165–10.341	0.8
Radiological findings				
	bilateral involvement	1.809	0.384–8.526	0.454
	cavitation	1.164	0.328–4.133	0.814
	effusion	0.507	0.064–4.007	0.519
Laboratory findings				
	Total Protein (g/dL)	0.589	0.290–1.198	0.144
	Albumin (g/dL)	0.41	0.176–0.954	0.038
	Cholinesterase (U/L)	0.992	0.982–1.002	0.106
	Hemoglobin (g/dL)	0.735	0.536–1.008	0.056
	CRP (mg/dL)	1.094	0.996–1.201	0.061
MUST score ≥ 2		4.401	0.557–34.742	0.16
MUST score ≥ 4		14.747	1.860–116.265	0.011

*HR*: hazard ratio; *CI*: confidence interval; *TB*: tuberculosis; *CRP*: C-reactive protein;

*MUST*: Malnutrition Universal Screening Tool.

**Table 4:** Multivariate Cox proportional hazard regression analyses associated with survival

**Variables**	**HR**	**95% CI**	**p-value**
Age (years)	1.088	1.017–1.165	0.015
MUST score ≥ 4	0.097	0.012–0.777	0.028

*HR*: hazard ratio; *CI*: confidence interval; *MUST*: Malnutrition Universal Screening Tool.

## Discussion

TB is often associated with nutritional deficiencies. A study of 30 patients with pulmonary TB in England revealed a reduction in BMI, triceps skin hold, arm muscle circumference and serum albumin [4]. In an Indian study, tuberculosis patients were respectively 11 and seven times more likely to have a BMI < 18.5 and mid-arm circumference < 24 cm [6].

The mortality rate among TB patients in Spain who completed treatment was found to be associated with vulnerable populations such as the elderly, alcohol abusers and HIV-infected injecting drug users [7]. We previously reported that subjective global assessment was useful in predicting the survival in patients with pulmonary tuberculosis [8]. In Japan, only 124 newly identified TB patients were reported as having HIV in 2007–2008 [2]. It seems that HIV infection does not have a large influence on the mortality rate among TB patients in Japan.

Patients with TB very frequently suffer from deficiencies of nutrients, such as vitamins A, B complex, C and E, as well as selenium, which are fundamental to the integrity of the immune response [9-11]. Three studies found lower levels of serum vitamin D in TB patients compared with those in controls [12-14]. Recently, Martineau et al. reported that vitamin D did not significantly affect time to sputum culture conversion in the whole study population, but it did significantly hasten sputum culture conversion in participants with the tt genotype of the TaqI vitamin D receptor during intensive-phase antimicrobial treatment of pulmonary tuberculosis [15]. In addition to the central role of lipid storage, adipose tissue has major endocrine functions and releases a variety of proinflammatory and anti-inflammatory factors, including adipocytokines, such as leptin, adiponectin and resistin, as well as cytokines and chemokines. Altered levels of different adipocytokines have been observed in a variety of inflammatory conditions and, in particular, the role of leptin in immune responses and inflammation has lately become increasingly evident [16]. Active tuberculosis is associated with cachexia, weight loss and low serum concentration of leptin [17,18]. Moreover, leptin-deficient mice are more susceptible to *M. tuberculosis* than wild-type mice, and T cell numbers, including those producing IFN-γ, are reduced in infected lungs, suggesting that leptin contributes to protection against tuberculosis [19]. However, a causative correlation between the severity of TB and leptin has not been fully established, and leptin concentrations do not predict wasting in human TB [18]. Nutrition and infection interact with each other synergistically. Recurrent infections lead to a loss of body nitrogen and worsen nutritional status; the resulting malnutrition, in its turn, produces a greater susceptibility to infection.

A number of nutrition screening tools have therefore been developed. MUST is a well-validated and reliable screening tool. Stratton et al. showed that MUST has predictive validity in an elderly hospitalized population, with regard to mortality, both in hospital and after discharge, and length of hospital stay [20]. Harris et al. reported that MUST was a sensitive and specific method of identifying those requiring further nutritional assessment in elderly people living in sheltered accommodation [21]. Recently, a cross-sectional study of the nutritional status of community-dwelling people with idiopathic Parkinson’s disease revealed the usefulness of MUST as an early screening tool [22].

Adoption of a cut-off value of 3.5 improves the predictive ability of MUST score as a prognostic indicator in pulmonary TB patients. MUST will be used in future studies of nutrition support in TB patients. One of the limitations in this study is the short duration of follow up. Minimum follow-up duration was 90 days. Other limitations of this study are the small sample size and imbalance of male/female ratio. For these reasons, it cannot be regarded as representative of the whole pulmonary TB population.

## Conclusion

MUST appears to be a reliable tool for nutritional risk assessment of patients with pulmonary TB. In addition, MUST may be a useful prognostic indicator of survival in patients with pulmonary TB.

## Abbreviations

TB: Tuberculosis; MUST: Malnutrition Universal Screening Tool; BMI: Body mass index; ROC: Receiver operating characteristic; AUC: Area under the curve; HR: Hazard ratio; CI: Confidence interval.

## Competing interests

The authors declare that they have no competing interests.

## Authors’ contributions

SM participated in the study design, acquisition of data, analysis and interpretation of data, and drafted the manuscript. MT participated in the study design and acquisition of data. DI participated in the study design, analysis and interpretation of data. All authors have read and approved the final manuscript.
